# Acanthamoeba castellanii alone is not a growth promoter for Hordeum vulgare

**DOI:** 10.1099/acmi.0.000761.v3

**Published:** 2024-08-01

**Authors:** Julia Sacharow, Stefan Ratering, Bellinda Schneider, Alessandra Österreicher Cunha-Dupont, Sylvia Schnell

**Affiliations:** 1 Institute of Applied Microbiology, IFZ, Justus-Liebig-University Giessen, 35392 Giessen, Germany

**Keywords:** *Acanthamoeba castellanii*, *Hordeum vulgare*, plant growth promoting, root architecture, soil food web, soil protozoa

## Abstract

Protists are important key players in the microbial loop and influence their environment by grazing, which leads to the return of nutrients into the soil and reduces pathogen pressure on plants. Specifically, protists on and around plant roots are important for plants’ development and growth. For this study, the fourth most important crop in the world, *Hordeum vulgare*, was selected. Seeds of *H. vulgare* were inoculated with *Acanthamoeba castellanii* alone or with additional soil bacteria at the beginning and during the experiment. The germination of the seeds and the growth of the plants in pouches were monitored over 3 weeks. No differences were found in leaf growth, root growth, root and leaf nitrogen content or ammonia content of the liquid from the pouches. In contrast, the relative increase in root and leaf dry weight showed a small difference compared to the controls. The results of this experiment demonstrated that seed inoculation with *A. castellanii* alone or with additional unidentified soil bacteria did not have a major effect on the growth and development of barley. Nevertheless, small changes in plant development were detected, indicating that *A. castellanii* should be considered for further investigation of co-inoculations with plant growth-promoting bacteria and additional nutrients.

**Impact Statement**

Implementing environmentally friendly approaches in agricultural farming is a crucial and significant challenge. Protists play a pivotal role in the growth, development and pathogenic pressure on plants. While several studies have explored the use of protists in promoting plant growth, the diverse range of essential agricultural plants has not received sufficient attention. This study is the first to focus on spring barley, one of the four major agricultural crops, and examines the use of protists to influence plant growth and development. The findings are of great importance to agricultural crop science and plant breeding, serving as a foundation for future applications of protists in agricultural crop production. The experiment reveal that not all plants respond similarly to protist treatment, emphasizing the need for further research to optimize this application for different plants.

## Data Summary

The authors confirm that all supporting data, code and protocols have been provided within the article or through supplementary data files. The figures and the supplementary material can be found in Figshare: https://doi.org/10.6084/m9.figshare.25962052.v1[1].https://microbiology.figshare.com/submit

## Introduction

Microorganisms are known to affect the growth and development of plants in their environment. Besides plant growth-promoting bacteria (PGPB) such as *Hartmannibacter diazotrophicus* E19 or *Bacillus amyloliquefaciens* NJN-6 [2,3] and arbuscular mycorrhizae fungi such as *Glomus mosseae* or *Glomus etunicatum* [4], protists are also known to influence root growth and plant performance [5]. In a now well-known process called the microbial loop, firstly described by Azam *et al*. (1983) [6] in aquatic systems and then in soils by Clarholm (1985) [7], plant species-specific protist communities graze on the microorganisms around the roots. The digestion of the microbial prey results in the release of nutrients into the rhizosphere and the uptake by the plant roots, promoting plant performance [8,9]. For this reason, protists are particularly well suited for agricultural formulations [10].

Jentschke *et al*. [11] reported that inoculated protists decreased the number of bacteria on the rhizoplane and positively influenced the growth of spruce seedlings. The authors concluded that the effect was not attributed to the release of nutrients by the grazing protists, as the concentration of mineral nutrients in the plant needles did not increase. Instead, they suggested that enhanced seedling growth was directly improved, following the production of phytohormones by the plant in the presence of protists, or indirectly, with the microeukaryotes altering the rhizosphere microbiome. Bonkowski and Brandt [12] further investigated these results. The process was later described as grazing-induced hormonal effects on root growth, where the selective grazing of protists favours indole-3-acetic acid (IAA)-producing plant growth-promoting bacteria [12,13]. While *Sphingobacteria*, *Rhodospirillales*, *Massilia*, *Caloramator* and, in particular, the plant growth-promoting bacterium *Azospirillum* sp. B510 benefit from protist grazing, other bacteria such as *Sphingomonadales*, *Ralstonia*, *Burkholderia* and *Rhodoferax* were reduced by the presence of inoculated protists [14]. In addition, the inoculation with the consortium of the plant growth-promoting bacteria *Azospirillum* sp. B510 and heterotrophic protists increased the plant growth and nitrogen uptake [14]. Recent research findings indicated that beneficial bacteria can even be transported within plant roots by protists [15]. Chandarana *et al*. [16] observed that the lateral roots, the seminal roots and the primary root length were significantly modified and that the N and P uptake was affected by a combined inoculation of ciliates and different plant growth-promoting bacteria. The age of the bacterial community is an important factor influencing this effect. Compared with the rhizosphere of younger plants, the percentage of IAA-producing bacteria in the rhizosphere of older plants decreased [17]. Other studies propose that soil nitrogen mineralization and the subsequent increase in nitrogen availability contribute to plant growth, as the proportion of IAA-producing bacteria in the community remained unaffected [18]. Zhang *et al*. [19] discovered that maize plant growth was improved when inoculated with the ciliate *Colpoda cucullus* and an added phosphate source. In addition, the grazing of protists had an influence on the reduction of the stress level of the plant, which was associated with shifts in the bacterial communities in the rhizosphere, as well as the increase of pathogen-suppressive microorganisms [20,22]. Inoculation with a consortium of protists can actually increase this effect [23].

The genus *Acanthamoeba* is often used for inoculation experiments. Kreuzer *et al*. [24] showed that the nitrogen balance of rice seedling roots grown in a small food web with soil bacteria and *Acanthamoeba castellanii* was 45 % higher than the control without the protist. Weidner *et al*. [25] showed that co-inoculation of beneficial bacteria and the amoeba *A. castellanii* resulted in a plant growth-promoting effect and reduced the harmful effects of a pathogen. It was also discovered that the grazing activity of *Acanthamoeba* on different plant growth-promoting bacteria led to positive but different developments in early rice plants [26].

In recent years, there has been increasing pressure to improve plant performance and ensure food security. Protists are essential for ecosystem functioning, promoting plant growth and improving plant health. As a result, they can form the basis of a new generation of environmentally friendly biological treatments, either as a stand-alone inoculant, in combination with plant growth-promoting bacteria or with additional nutrition sources. This study seeks to determine whether *A. castellanii*, when used as a stand-alone inoculant, can positively influence the root growth and overall performance of *Hordeum vulgare*.

## Methods

### *A. castellanii* culture and soil bacteria

The *A. castellanii* culture 1534/3, with an unidentified prey mixture of *Escherichia coli* and various other bacteria, was ordered from Culture Collection of Algae and Protozoa (Oban, UK). The protist was grown in Petri dishes (Ø 14.5 cm, 83.3903, Sarstedt AG and Co. KG, Germany) with a mixture of 5 ml of 0.5 % lysogeny broth medium (Carl Roth, Germany) and 100 ml of autoclaved Volvic water (Danone, France). After 7 days, the protist culture was viewed under the microscope at 630× magnification (DM 1000 LED, Leica, Germany; RRID:SCR_020225) and cleared of bacteria once by centrifugation at 1000 g for 10 min (Megafuge 1.OR, Thermo Fischer Scientific, USA) modified according to Kreuzer *et al*. [24]. The protists’ concentration in the purified *A. castellanii* culture, the concentration of remaining bacteria in the purified *A. castellanii* culture and the bacterial concentration in the supernatant from the purification step were counted using a haemocytometer (Neubauer improved 0.02 mm, Hecht-Assistant, Germany).

A bacterial culture was obtained from organic farm soil from the Gladbacherhof (50° 23′ N, 8° 15′ E). The soil was filled into sterile 100 ml glass bottles and covered with autoclaved Volvic water. Soil suspensions were mixed for 10 min at 100 rpm on an overhead shaker (Reax 2 Heidolph Instruments, Germany) and left at room temperature for 3 days. After 3 days, the water above the soil was filtered through a filter paper (3 µm; Whatman, UK) using a vacuum pump (MZ 2C NT, Vacuubrand, Germany) to collect a (soil) bacterial suspension free of protists. The bacterial suspension was checked for protists, and the bacterial number was counted using the haemocytometer.

### Experimental setup

Different treatments with *A. castellanii* alone or with additional soil bacteria were prepared for analysis. The surface of the *H. vulgare* ‘Odilla’ seeds was sterilized according to the protocol of Garcia *et al*. [27]. In detail, the seeds were filled in a sterile glass bottle, covered with a 3 % chlorine solution and shaken for 2 min. After discarding the chlorine solution, the seeds were covered with sterile deionized water and shaken for 1 min. This step was repeated five times. The following step entailed inoculating the seeds with the different treatments. A concentration of 10^5^ protists per ml was used for the protist treatments. For the bacterial treatments, a concentration of 10^6^ bacteria per ml was used, based on the concentration of remaining bacteria in the purified *A. castellanii* culture that had been previously counted. The surface-sterilized seeds were combined with 20 ml of the inoculum culture in a sterile 50 ml plastic centrifuge tube (Sarstedt AG and Co. KG, Germany). The mixture was then shaken at 100 rpm on an orbital shaker (PSU-20i, Bio San, Latvia) at 25 °C for 1 h. After inoculation, the seeds were distributed in autoclaved growth pouches (maximum capacity 620 ml, Mega International, USA) with five seeds per pouch, as described below (Table 1) and filled with the following solutions to create an aqueous growth system. For treatments 1, 3 and 5, 30 ml of autoclaved Volvic water and 3 ml of soil bacterial solution were added into the pouches, and for treatments 2, 4 and 6, 33 ml of autoclaved Volvic water were added. To maintain the humidity, the pouches were covered with new plastic bags. A total of 100 seeds were allocated per treatment. The pouches were placed in a growth chamber in the dark at 25 °C for 6 days to initiate seed germination. After 6 days, the plastic bags were removed, and the pouches were placed in a greenhouse at 20 °C and 10 kLx for 16 h and 14 °C without artificial light for 8 h.

**Table 1.** Overview of the two treatments and their individually suitable controls

Treatment	Treatment no.	Suitable controls	Control no.
***A. castellanii* with soil bacteria**	(1)	Supernatant of *A. castellanii* with soil bacteriaVolvic water with soil bacteria	(3)(5)
***A. castellanii***	(2)	Supernatant of *A. castellanii*Volvic water	(4)(6)

### Sampling and re-treating

After 1 day in the greenhouse, in total 7 days after the experiment started, the first plants were sampled. The roots of 15 plants per treatment were measured with the WinRHIZO Pro 2019 (Regent Instruments Inc., Canada; RRID:SCR_017120), the longest leaf was measured, a liquid sample was taken and the fresh weight was determined (LS 620M SCS, Precisa, Switzerland). The whole leaf and root material was dried at 105 °C for 24 h in a drying cabinet (ULE 500, Memmert, Germany) and milled in a Retsch mill (MM400, Retsch GmbH, Germany; RRID:SCR_020427). In detail, the samples were placed in 2 ml cups (Sarstedt AG and Co. KG, Germany), small and large iron balls were added, and the cups were shaken for 10 min at 30 s^−1^. The relative biomass increase was calculated by transforming the response ratio into a percentage relative increase [28]. For the C/N measurement, samples were filled in small tin boats (4×4×11 mm, Elementar Analysensysteme, Germany). For plant material, 5–7 mg was placed in the boats, and for root material, 10 mg was placed in the boats. The filled boats were compressed and subjected to elemental analysis (Unicube, Elementar, Germany). Part of the liquid sample was used to determine the ammonia concentration with the method of Kandeler and Gerber [29] after the extraction with 1 M KCl (Roth, Germany), and the other part was analysed under the microscope. The concentration of *A. castellanii* was determined by counting with the haemocytometer. Because bacterial and fungal contaminations were not the focus of this study, their density was estimated only. This assessment was qualitative, based on the density of the cells and classified as low density, dense and very dense.

The remaining plants in the greenhouse were re-inoculated with microorganisms of the corresponding treatments to ensure that there were always enough active trophozoites in contact with the roots. Cultures were prepared as described above, and 50 ml of autoclaved Volvic water and 10 ml of the cultures were added to the pouches. In addition, 3 ml soil bacterial solution was added to treatments 1, 3 and 5, and 3 ml of autoclaved Volvic water was added to treatments 2, 4 and 6. Seven days later (in total 14 days after the experiment started), the second sampling took place, and the remaining plants in the greenhouse were treated as described above. The final sampling was done after a further 7 days on site (in total 21 days after the experiment started), as described above.

### Statistical analysis

Statistical analysis was performed using R version 4.0.3 [30] (RRID:SCR_001905). The data was tested for normal distribution using Shapiro–Wilk’s test [31] and for homogeneity of variances using the Bartlett test [32]. For data with a normal distribution and homogeneity of variances, statistical tests were performed using ANOVA [33] (RRID:SCR_002427) and a post-hoc pairwise *t*-test [34] (*P*<0.05). When data were not normally distributed, analyses were done with a non-parametric Kruskal–Wallis test [35] and a post-hoc pairwise Wilcoxon rank-sum test [36] (*P*<0.05). In the case of heteroscedasticity, the data were analysed using a Welch ANOVA followed by a post-hoc pairwise *t*-test (*P*<0.05). For the pairwise comparisons between the groups, the *P*-values were multiplied by the number of comparisons according to the Benjamini and Hochberg adjustment method [37]. The script can be found in the supplementary material. The figures were prepared with Origin 2017 (RRID:SCR_014212) [38] and R (package ggstatsplot version 0.12.1 [39]).

## Results

### Visual classification of plant growth and development

**Fig. 1.**
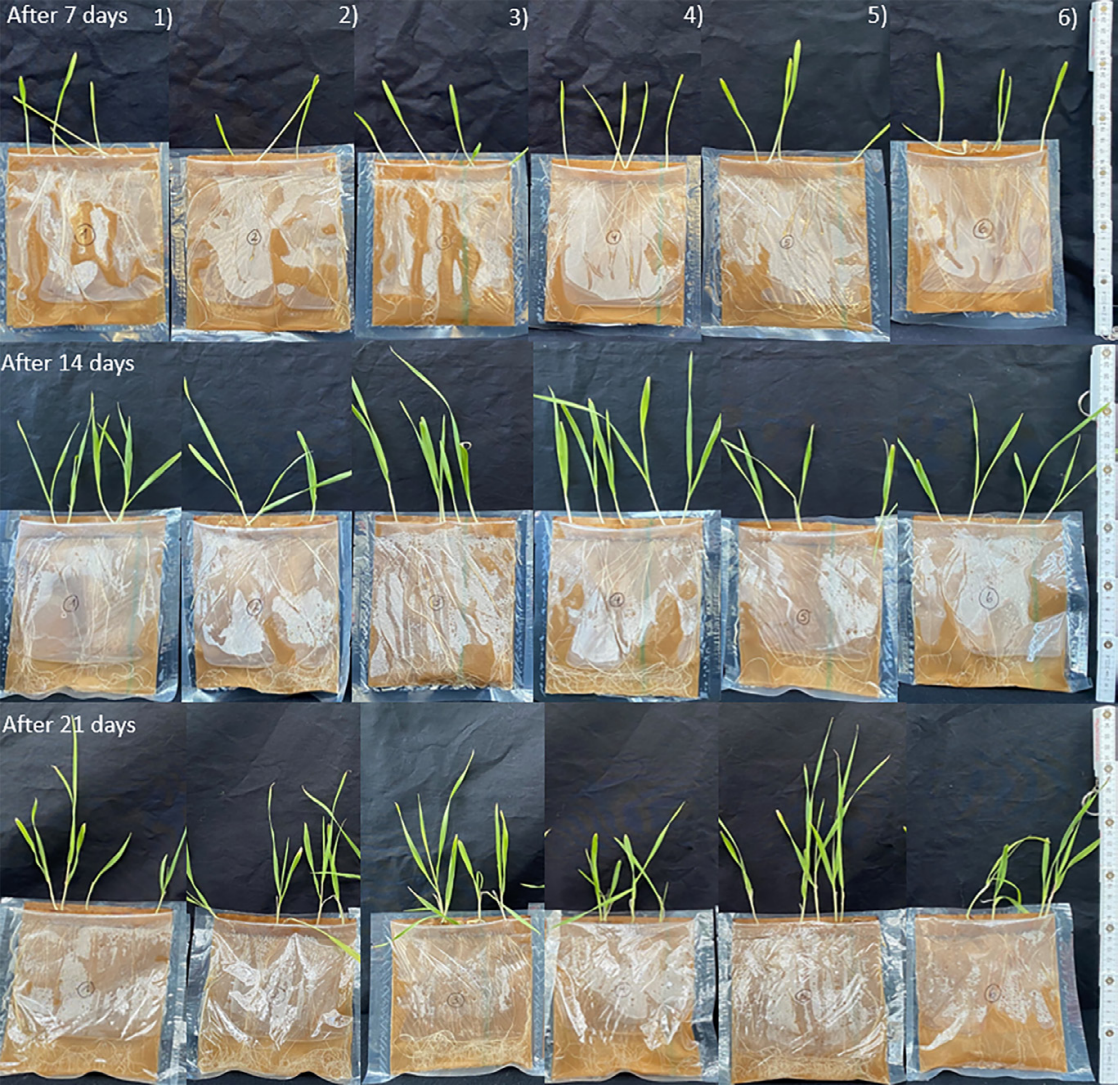
Barley plants at 7, 14 and 21 days after the start of the experiment. The different treatments are arranged per column: (1) *A. castellanii* with soil bacteria, (2) *A. castellanii*, (3) Supernatant of *A. castellanii* with soil bacteria, (4) Supernatant of *A. castellanii*, (5) Volvic water with soil bacteria and (6) Volvic water.

No visible differences in root or leaf growth were observed at sampling at 7, 14 and 21 days after the start of the experiment for all barley plant treatments in the pouches (Fig. 1). The only detectable differences were in length, due to natural plant growth, between sampling times.

### Root growth analysis

**Fig. 2.**
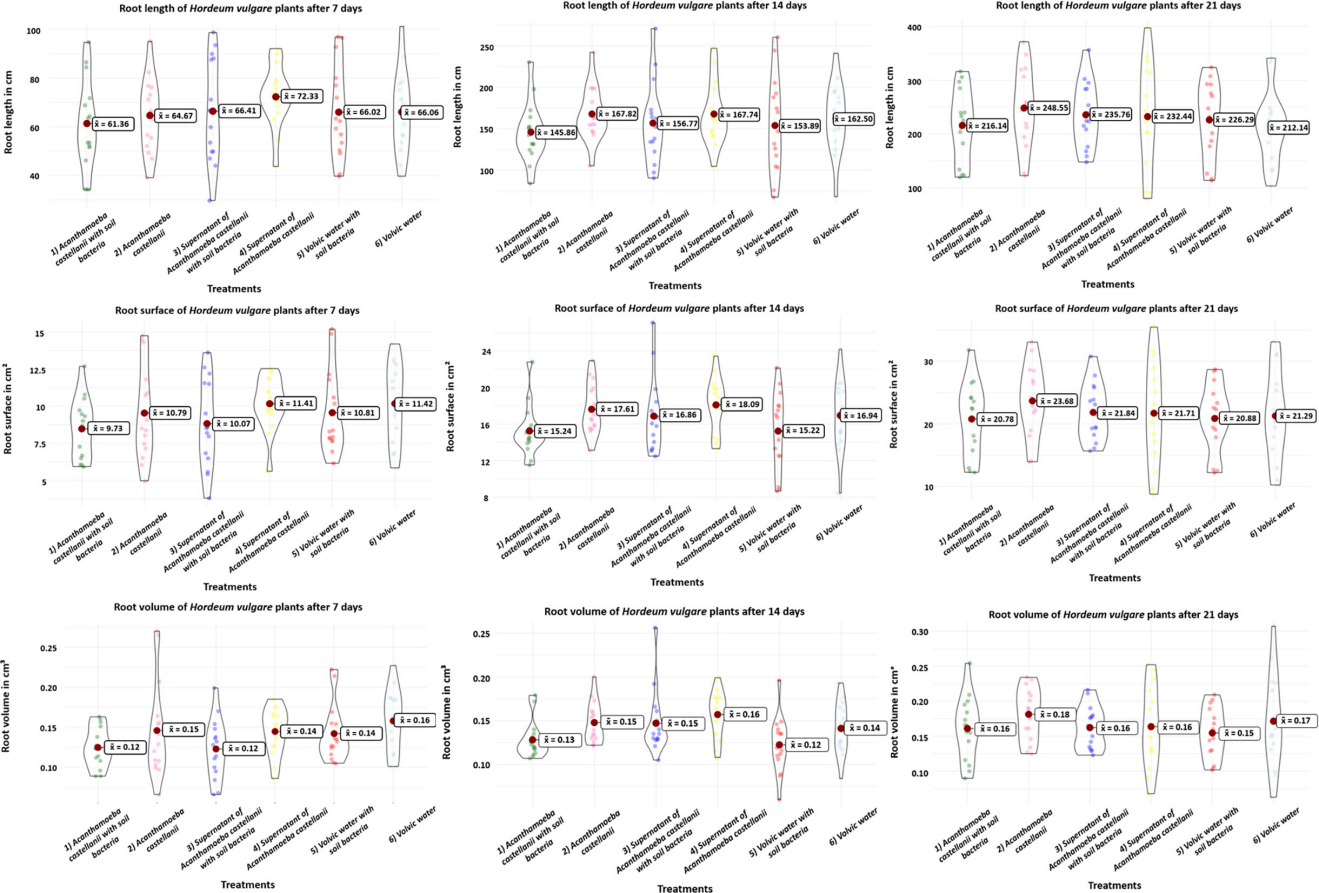
Root growth (summed length in cm, surface in cm^2^ and volume in cm^3^, in a column) of the barley plants at sampling points at 7, 14 and 21 days (in a row) after the start of the experiment. Each plot contains treatments (1) *A. castellanii* with soil bacteria, (2) *A. castellanii*, (3) Supernatant of *A. castellanii* with soil bacteria, (4) Supernatant of *A. castellanii*, (5) Volvic water with soil bacteria and (6) Volvic water. The symbol $\bar{x}$ describes the mean value.

Root growth measurements (length, surface and volume) showed no visible differences (Fig. 2, overview Table 2, standard deviation *σ* – Table S1, available in the online Supplementary Material). At the first sampling, the treatment (4) Supernatant of *A. castellanii* had the longest summed roots with an average of 72.33 cm, and the treatment (1) *A. castellanii* with soil bacteria had the smallest roots with an average of 61.36 cm. Root surface measurements showed the largest summed mean root surface for the treatment (6) Volvic water and the smallest for the treatment (1) *A. castellanii* with soil bacteria. Similarly, the treatment (6) Volvic water had the largest summed mean value for root volume, while treatments (1) *A. castellanii* with soil bacteria and (3) Supernatant of *A. castellanii* with soil bacteria had the lowest summed root volumes. At the second sampling, the treatment (2) *A. castellanii* had the longest average summed roots and the treatment (1) *A. castellanii* with soil bacteria had the shortest. At the third sampling, the treatment (2) *A. castellanii* had the longest average summed roots and the treatment (6) Volvic water had the shortest. No specific treatment appeared to consistently enhance root growth in terms of length, surface area or volume. Furthermore, the difference between the highest and lowest measured values was generally small. Statistical analyses showed no significant differences between any treatment, at any time point, for any of the measured root growth parameters (root length, root surface and root volume) (Table S2).

**Table 2.** Overview of the highest and lowest measured mean values for root length, root surface and root volume of 15 plants at days 7, 14 and 21, for treatments (1) *A. castellanii* with soil bacteria, (2) *A. castellanii*, (3) Supernatant of *A. castellanii* with soil bacteria, (4) Supernatant of *A. castellanii*, (5) Volvic water with soil bacteria and (6) Volvic water

	Highest value	Treatment with highest value	Lowest value	Treatment with lowest value
Root length in cm after 7 days	72.33 cm	(4)	61.36 cm	(1)
Root length in cm after 14 days	167.82 cm	(2)	145.85 cm	(1)
Root length in cm after 21 days	235.76 cm	(2)	212.14 cm	(6)
Root surface in cm^2^ after 7 days	11.42 cm^2^	(6)	9.73 cm^2^	(1)
Root surface in cm^2^ after 14 days	18.09 cm^2^	(4)	15.22 cm^2^	(5)
Root surface in cm^2^ after 21 days	23.68 cm^2^	(2)	20.78 cm^2^	(1)
Root volume in cm^3^ after 7 days	0.16 cm^3^	(6)	0.12 cm^3^	(3)
Root volume in cm^3^ after 14 days	0.16 cm^3^	(4)	0.12 cm^3^	(5)
Root volume in cm^3^ after 21 days	0.18 cm^3^	(2)	0.15 cm^3^	(5)

### Leaf length analysis

**Fig. 3.**
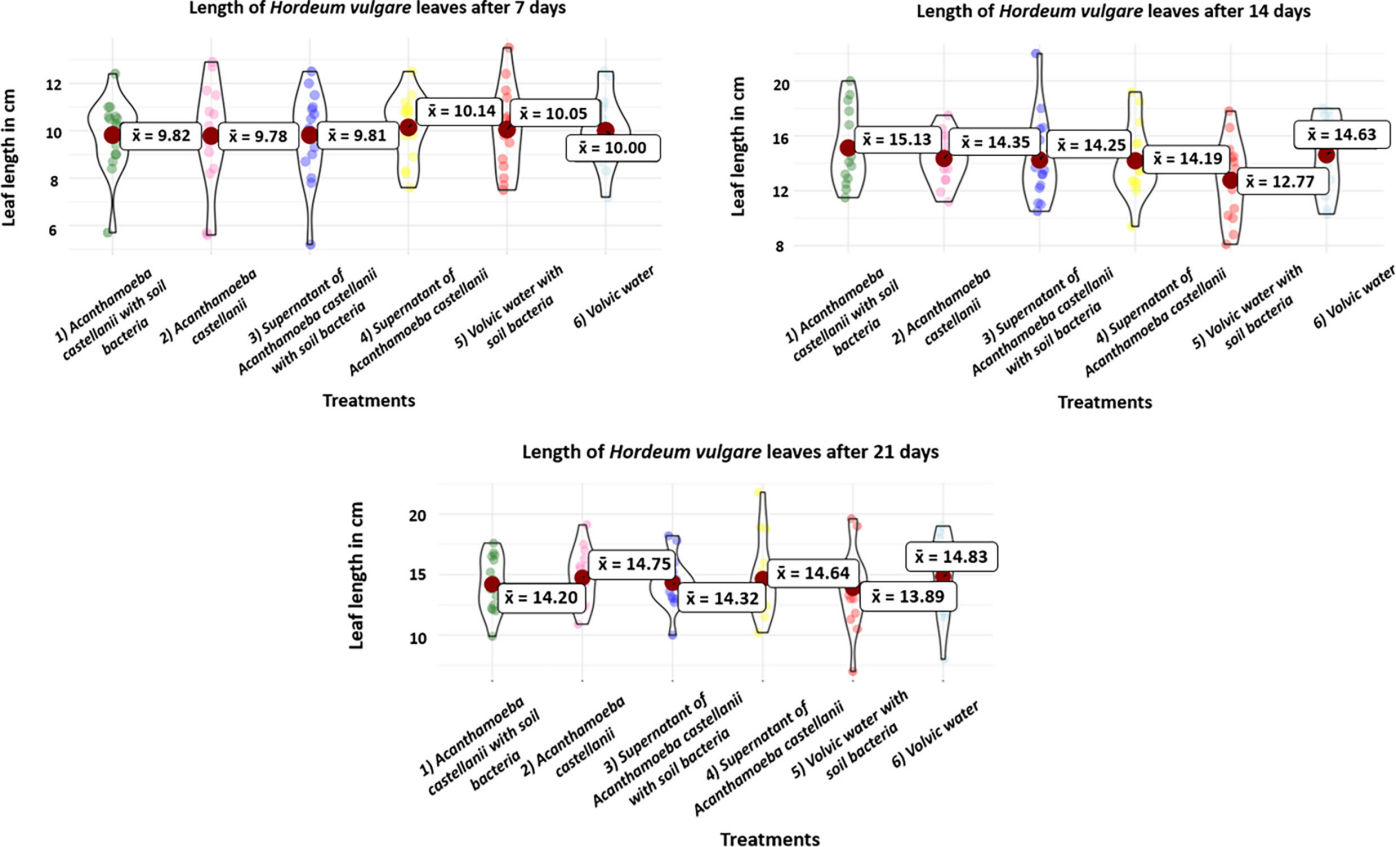
Leaf length in cm of the barley plants at the different sampling points at 7, 14 and 21 days after the start of the experiment. With treatments (1) *A. castellanii* with soil bacteria, (2) *A. castellanii*, (3) Supernatant of *A. castellanii* with soil bacteria, (4) Supernatant of *A. castellanii*, (5) Volvic water with soil bacteria and (6) Volvic water. The symbol $\bar{x}$ describes the mean value.

Leaf length measurements showed no visible or significant differences between the treatments (Fig. 3, standard deviation σ – Tables S3 and S4). At the first sampling, the treatment (4) Supernatant of *A. castellanii* showed the longest leaf average 10.14 cm and the treatment (2) *A. castellanii* showed the smallest with 9.78 cm. At the second sampling, the treatment (1) *A. castellanii* with soil bacteria showed the longest leaf average and the treatment (5) Volvic water with soil bacteria showed the smallest. At the third sampling, the treatment (4) Supernatant of *A. castellanii* had the longest leaf average and the treatment (5) Volvic water with soil bacteria had the smallest. There was no apparent consistency in the highest and lowest measured values of leaves over time, i.e. no treatment, in particular, seems to promote or reduce leaf growth. Additionally, the distinction between the highest and lowest values was generally small.

### Fresh, dry weight and relative increase of leaf and root material

**Fig. 4.**
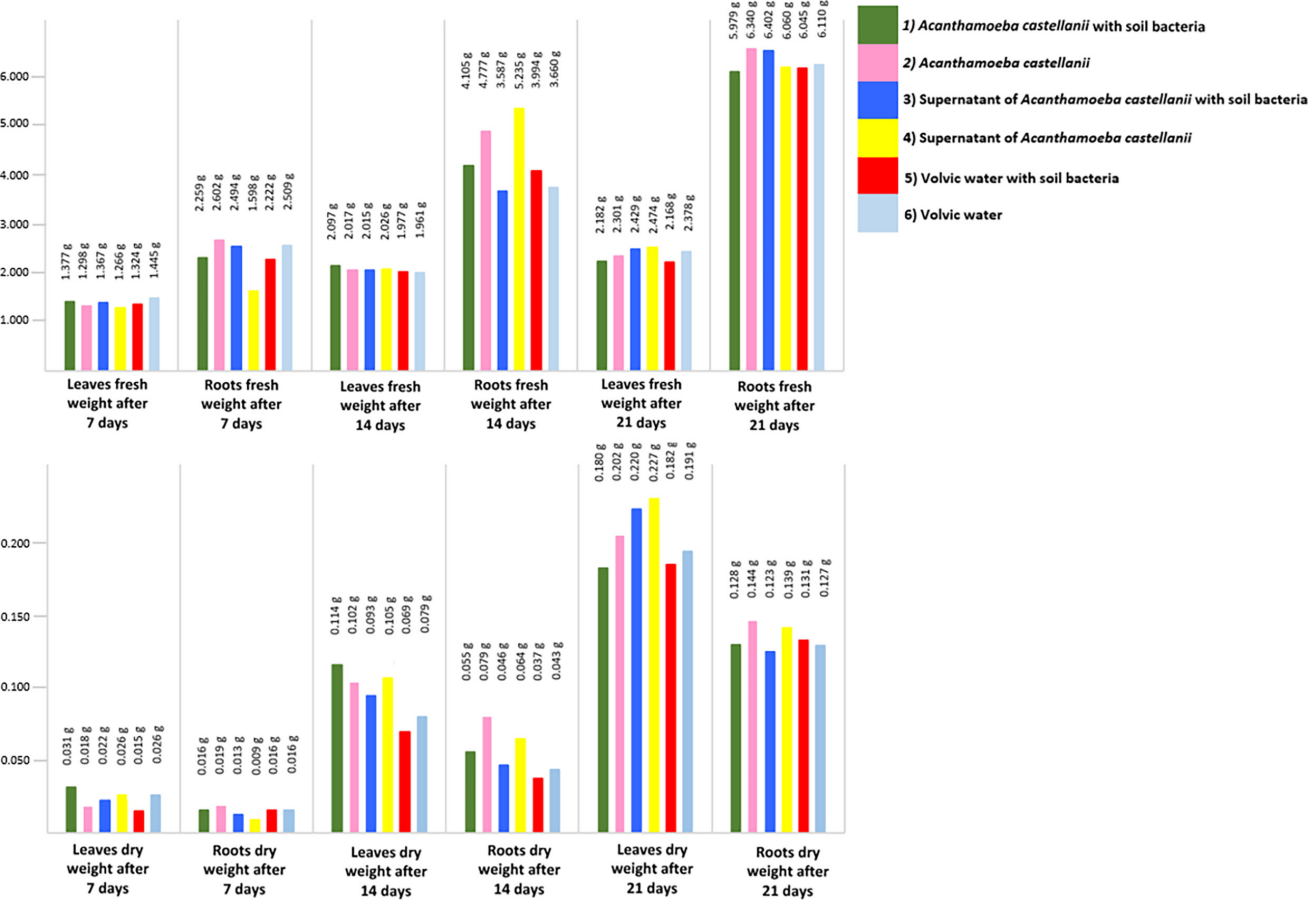
Fresh weight (above) and dry weight (below) given in grams for treatments (1) *A. castellanii* with soil bacteria (green), (2) *A. castellanii* (pink), (3) Supernatant of *A. castellanii* with soil bacteria (blue), (4) Supernatant of *A. castellanii* (yellow), (5) Volvic water with soil bacteria (red) and (6) Volvic water (light blue). Barplots of whole leaf and root material (15 plants, no replicates) at 7, 14 and 21 days after the start of the experiment. Sampling times are grouped per row.

Fresh and dry weight values, as well as a relative increase (RI) [28] in the biomass and water content (difference between fresh and dry biomass) of the whole leaf and root material (15 plants together, no replicates, as the roots were too small for individual analyses at the beginning), showed some differences at the three sampling points. After 7 days, the treatment (6) Volvic water showed the highest leaf fresh weight value and the treatment (2) *A. castellanii* showed the lowest. Moreover, leaf dry weight was the highest in the treatment (1) *A. castellanii* with soil bacteria, while the treatment (3) Supernatant of * A. castellanii* with soil bacteria showed the lowest value. The same kind of variation was detected for the second sampling. At the third sampling, the treatment (4) Supernatant of *A. castellanii* showed the highest value and the treatment (1) *A. castellanii* with soil bacteria showed the lowest for the leaf dry weight. Similar variations were detected for fresh and dry root weight. At the first sampling, the treatment (2) *A. castellanii* showed the highest fresh weight and the treatment (4) Supernatant of * A. castellanii* showed the lowest. The treatments (1) *A. castellanii* with soil bacteria, (5) Volvic water with soil bacteria and (6) Volvic water had the highest root dry weight and the treatment (4) Supernatant of *A. castellanii* had the lowest. The same kind of variation was detected for the second sampling. At the third sampling, the treatment (2) *A. castellanii* showed the highest root dry weight, while the treatment (3) Supernatant of *A. castellanii* with soil bacteria showed the lowest value (Fig. 4).

Fluctuations were observed in the relative increase of dry weight in leaf and root materials, with treatments exhibiting values both higher and lower than their respective controls (Table 3). From the first to the last sampling, the values for the relative increase in dry weight of leaves in treatment (1) *A. castellanii* with soil bacteria, compared with the treatment (3) Supernatant of *A. castellanii* with soil bacteria, decreased from 40.9 to 18.2 %. The highest values for the relative increase in dry weight of roots from the treatment (1) *A. castellanii* with soil bacteria were observed at the second sampling. In contrast to the first sampling where the relative increase was 0 % compared with the (5) Volvic water with soil bacteria, it showed a 48.6 % change at the second sampling. The treatment (2) *A. castellanii* mostly showed a negative relative increase in dry weight of leaves. However, comparing the treatments (2) *A. castellanii* and (4) Supernatant of *A. castellanii*, the relative increase in dry weight of roots was consistently positive, reaching 111.1 % and decreasing to 3.6 % at the third sampling.

**Table 3.** Relative increase (RI) of biomass and water content (difference between fresh and dry weight) of the whole leaf and root material (15 plants, no replicates). (A) Comparison between the treatment (1) *A. castellanii* with soil bacteria and controls (3) Supernatant of *A. castellanii* with soil bacteria and (5) Volvic water with soil bacteria. (B) Comparison between the treatment (2) *A. castellanii* and controls (4) Supernatant of *A. castellanii* and (6) Volvic water, at 7, 14 and 21 days after the start of the experiment

(A)						
	**First sampling**		**Second sampling**		**Third sampling**	
(1) *A. castellanii* with soil bacteria in comparison to	(3) Supernatant of *A. castellanii* with soil bacteria	(5) Volvic water with soil bacteria	(3) Supernatant of *A. castellanii* with soil bacteria	(5) Volvic water with soil bacteria	(3) Supernatant of *A. castellanii* with soil bacteria	(5) Volvic water with soil bacteria
RI biomass leaves (%)	40.9	106.7	22.6	65.2	−18.2	−1.1
RI biomass roots (%)	23.1	0	19.6	48.6	4.1	−2.3
RI water content leaves (%)	0.01	2.8	3.2	3.9	−9.4	0.8
RI water content roots (%)	−9.6	1.7	14.4	2.4	−50.6	−1.1

(B)						
(2) *A. castellanii* in comparison to	(4) Supernatant of *A. castellanii*	(6) Volvic water	(4) Supernatant of *A. castellanii*	(6) Volvic water	(4) Supernatant of *A. castellanii*	(6) Volvic water
RI biomass leaves (%)	−30.8	−30.8	−2.9	29.1	−11	5.8
RI biomass roots (%)	111.1	18.8	23.4	83.7	3.6	13.4
RI water content leaves (%)	3.2	−9.2	−0.3	1.8	−6.6	−4.0
RI water content roots (%)	62.6	3.6	−9.1	29.9	6.2	5.1

### Viability of the inoculated protists in the pouches

After each sampling, the liquid from the pouches was examined under a microscope to check the viability of the inoculated *A. castellanii* (Table 4). The liquid from the first sampling showed that *A. castellanii,* inoculated with soil bacteria(2) or alone (3), survived in the pouches as trophozoites and cysts. The protists’ trophozoites were mobile and had a concentration of 10^5^ per ml in both treatments. The bacterial density differed between the two treatments: the treatment (1) *A. castellanii* with soil bacteria had a dense bacterial suspension, while the treatment (2) *A. castellanii* had a low bacterial density. The treatment (3) Supernatant of *A. castellanii* with soil bacteria showed high bacterial density, similar to the treatment (5) Volvic water with soil bacteria, while the treatment (4) Supernatant of *A. castellanii* showed low bacterial density, just like the treatment (6) Volvic water. The liquid from the second sampling showed that the *A. castellanii* inoculated with soil bacteria (1) and alone (2) was still alive and mobile and had a concentration of 10^5^ per ml in both treatments. The bacteria were equally dense in the two treatments. The treatments (3) Supernatant of *A. castellanii* with soil bacteria, (4) Supernatant of *A. castellanii* and (5) Volvic water with soil bacteria showed a dense bacterial presence and a low fungal contamination. In parallel, the treatment (6) Volvic water had a low density of bacterial and fungal contaminations. At the last sampling, the sampled liquid from the pouches showed that the *A. castellanii* inoculated with soil bacteria (1) and alone (2) was still alive and mobile in the trophozoite form, with a concentration of 10^5^ per ml in both treatments. Bacterial density was higher in the treatment (2) than in the treatment (1), and the treatment (2) had a fungal contamination. All control treatments had very dense bacterial populations and fungal contaminations. A picture of *A. castellanii* from the treatment (1) *A. castellanii* with soil bacteria can be found on the supplement (Fig. S1). No additional pictures were captured as they did not exhibit any remarkable features.

**Table 4.** Classification (counted concentration of *A. castellanii* and estimated density of bacterial and fungal contaminations) of the liquid from the pouches at the three different sampling points (7, 14 and 21 days after the start of the experiment) from the treatments (1) *A. castellanii* with soil bacteria, (2) *A. castellanii*, (3) Supernatant of *A. castellanii* with soil bacteria, (4) Supernatant of *A. castellanii*, (5) Volvic water with soil bacteria and (6) Volvic water

	First sampling	Second sampling	Third sampling
(1) *A. castellanii* with soil bacteria	Trophozoites and cysts of *A. castellanii* with a concentration of 10^5^ per ml; dense bacterial presence	Trophozoites and cysts of *A. castellanii* with a concentration of 10^5^ per ml; dense bacterial presence	Trophozoites and cysts of *A. castellanii* with a concentration of 10^5^ per ml; dense bacterial presence
(2) *A. castellanii*	Trophozoites and cysts of *A. castellanii* with a concentration of 10^5^ per ml; low bacterial density	Trophozoites and cysts of *A. castellanii* with a concentration of 10^5^ per ml; dense bacterial presence	Trophozoites and cysts of *A. castellanii* with a concentration of 10^5^ per ml; very dense bacterial and fungal presence
(3) Supernatant of *A. castellanii* with soil bacteria	Dense bacterial presence	Dense bacterial presence and low fungal density	Very dense bacterial and fungal presence
(4) Supernatant of *A. castellanii*	Low bacterial density	Dense bacterial presence and low fungal density	Very dense bacterial and fungal presence
(5) Volvic water with soil bacteria	Dense bacterial presence	Dense bacterial presence and low fungal density	Very dense bacterial and fungal presence
(6) Volvic water	Low bacterial density	Low bacterial and fungal density	Very dense bacterial and fungal presence

### Nitrogen and ammonium content

**Fig. 5.**
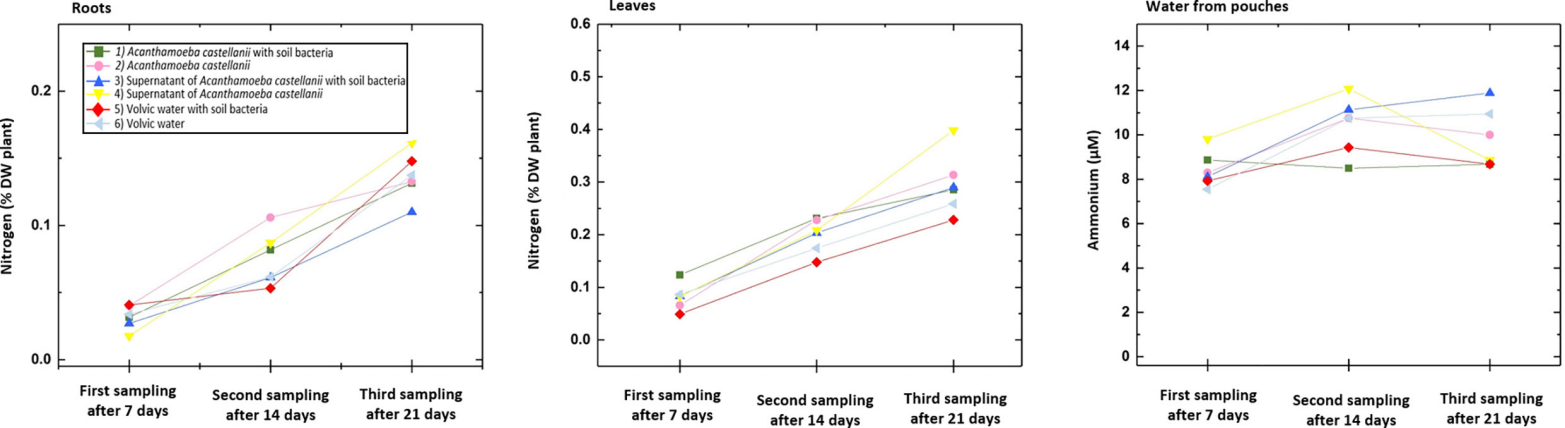
Nitrogen content in % of plant dry weight (DW) of the dried roots (left), leaves (middle) and ammonium content in µM of the liquid from the pouches (right) after 7, 14 and 21 days after the beginning of the experiment. The treatment (1) *A. castellanii* with soil bacteria (green) and the control treatments (3) Supernatant of *A. castellanii* with soil bacteria (blue) and (5) Volvic water with soil bacteria (red) as well as the treatment (2) *A. castellanii* (pink) with the control treatments (4) Supernatant of *A. castellanii* (yellow) and (6) Volvic water (light blue).

The nitrogen content of dried roots and leaves at all three sampling points exhibited no variations between any of the treatments and the controls (Fig. 5, left and middle). At the first sampling, the treatment (5) Volvic water with soil bacteria showed the highest measured value, while the treatment (4) Supernatant of *A. castellanii* showed the lowest one. All measured values were between 0.0 and 0.05 % dry weight (DW) plant. The nitrogen content of the leaves ranged between the treatment (4) Supernatant of *A. castellanii* with the highest value and the treatment (1) *A. castellanii* with soil bacteria with the lowest value. At the second sampling, apart from treatment (5) Volvic water with soil bacteria (leaf nitrogen content), no discernible consistency in the highest and lowest nitrogen concentrations was observed compared to the first sampling. The same pattern was observed at the third sampling. The nitrogen content of the roots fluctuated between the treatment (4) Supernatant of * A. castellanii* with the highest value and the treatment (3) Supernatant of *A. castellanii* with soil bacteria with the lowest value. There was no visible consistency in the highest and lowest nitrogen content over time, i.e. no particular treatment appears to promote a high nitrogen content in leaf or root material.

The ammonia content of the liquid from the pouches showed no differences between any of the treatments and controls at all three sampling points (Fig. 5, right). At the first sampling, the ammonium content fluctuated between the treatment (6) Volvic water with the lowest measured value and the treatment (4) Supernatant of *A. castellanii*. No discernible consistency was observed in the treatment with the highest and lowest ammonium content during the second and third samplings.

The figures and the supplement can be found in Figshare: https://doi.org/10.6084/m9.figshare.25962052.v1 https://microbiology.figshare.com/submit[1].

## Discussion

The results of the experiment showed that the growth of barley plants was only slightly influenced by the presence of * A. castellanii*, with or without additional soil bacteria. There were no significant differences in leaf or root growth between treated and control plants (Figs.1^3^ and Tables S2 and S4). At each sampling point, different treatments displayed both the highest and lowest growth rates, indicating inconsistent growth patterns across treatments. Additionally, the growth difference between the best and worst treatments at any given time point was generally minor. Nevertheless, some slight influences were seen by the relative increase of leaf and root dry material (Fig. 4). Although *A. castellanii* was reinoculated after each sampling and the microscopy results showed that the *A. castellanii* survived in both treatments, only some small, non-significant differences could be detected in the experiment.

This experiment demonstrated that inoculating barley seeds with *A. castellanii* alone or in combination with an unidentified mixture of soil bacteria did not adequately enhance barley growth. Kreuzer *et al*. [24] showed that the inoculation of rice seedlings with * A. castellanii* and soil bacteria affected root growth and increased root nitrogen content. The rice shoots grown in the presence of * A. castellanii* and soil bacteria contained 45 % more nitrogen than the control roots. In barley seeds, inoculation with the amoeba did not affect root growth and nitrogen content. Instead, the differences between the treatments at the three sampling points were marginal. Small treatment effects were only observed regarding the dry weight of the root material from the first two samplings, and no effects could be seen or measured for plant growth or root numbers, similar to the findings of Kreuzer *et al*. [24]. Because Kreuzer *et al*. [24] did not have a control treatment with soil bacteria, and without *A. castellanii*, it is possible that the complex system of non-identified soil bacteria strongly influenced the dry weight by providing IAA-producing bacteria, for example. The composition of the soil bacterial community in the pouches depends on several different factors, such as the sampling site from which the soil was taken for isolation, the process of preparing these bacteria and the experimental growth conditions in the test. This makes reproducibility difficult, as the numbers and types of PGPR present can vary greatly. Jentschke *et al*. [11] and Krome *et al*. [40] hypothesized that protists indirectly affect plant growth by consuming non-IAA-producing bacteria, reducing the competitor bacteria, thus allowing the IAA-producing bacteria to be more active and hereby activating plant metabolism and the production of plant phytohormones. Chandarana *et al*. [16] demonstrated that co-inoculating ciliates with IAA-producing and nitrogen-fixing bacteria, the grazing of ciliates on the bacteria resulted in increased nitrogen and phosphate uptake and improved root growth of rice seedlings grown in soil. Furthermore, the inoculation with protists also influenced the plant growth-promoting attributes of bacteria [41]. In another study, Chandarana *et al*. [26] also demonstrated that the co-inoculation and grazing of *Acanthamoeba* on plant growth-promoting bacteria affect the root growth of rice seedlings grown on agar better than the inoculation of plant growth-promoting bacteria without *Acanthamoeba*. As no significant effects have been measured in this experiment, it can be assumed that the amount of naturally occurring IAA-producing and nitrogen-fixing bacteria in the soil bacteria from the treatment (1) *A. castellanii* with soil bacteria was maybe too low, even though the soil was from an organic farm. Based on the literature results, it can be concluded that co-inoculating *A. castellanii* with nitrogen-fixing plant growth-promoting bacteria or other protists [23] could lead to better results and plant growth. The utilization of rice seedlings in all experiments by Kreuzer and Chandarana, coupled with their observation of the impact of co-inoculated protists on root development, implies that the enhancement in plant root growth is likely attributable not only to the co-inoculation itself but also to the sensitivity and uptake mechanisms of the plant roots. Roots are the most sensitive part of a plant, but the sensitivity of roots varies. Su *et al*. [42] showed in their experiment that the roots of rice plants have a higher arsenite uptake than wheat and barley. A higher uptake of additional nutrients from the grazing protists could lead to the plant growth effect observed in these experiments. Besides the missing nitrogen-fixing and IAA-producing bacteria and the differences in root uptake, a missing nutrient source could explain the results and the lack of plant growth. The small differences in the relative increase of dried material are higher in the first two samplings than in the third sampling. Especially at the first sampling, the nutrients from the germinated seeds as well as the added *A. castellanii* could trigger the barley plants and lead to a difference in the relative increase of dry weight of 106.7 % for leaf and 111.1 % for root material. Zhang *et al*. [19] demonstrated that co-inoculating ciliates with various phosphate sources increased the phosphate availability in both soil and plants, leading to higher dry weights. The inoculated *A. castellanii* could affect the uptake and processing of nutrients by the seeds. By the third sampling point, the nutrient reserves of the seeds may have been depleted, and the relative increase in dry weight from the treatments equalized. In addition to these positive findings, Berlinches *et al*. [23] also reported some negative outcomes. They found that inoculating with protist consortia helped plants reduce pathogenic stress and partially increased biomass, but the effects were not consistent or significant in all cases. This experiment showed that inoculating barley seeds with the protist *A. castellanii* alone or with an unidentified soil bacteria mix did not influence barley seed growth, in contrast to the literature results for rice plants. Because some differences in dry weight could be observed at the beginning of the experiment, optimizing the co-inoculation of barley seeds with *A. castellanii* and additional nutrients and/or nitrogen-fixing IAA-producing bacteria could be a good approach to promote barley plants’ growth since the use of environmentally friendly plant-growth products are important for the future of food production, and protists could be suited to agricultural usage [10].

## Conclusion

Protists are good candidates for environmentally friendly crop production and plant growth promotion. The model organism * A. castellanii*, which is often used for experiments, showed potential for use as a biological inoculant to promote plant growth. In this study, the inoculation with *A. castellanii* with or without additional soil bacteria showed no differences in leaf growth, root growth, nitrogen content and ammonia content compared to the controls. Nevertheless, the relative increase in dry weight from the inoculated plants showed some differences compared to the controls at the beginning of the experiment. Based on these results and the literature, inoculating barley seeds with *A. castellanii* alone or with an unidentified mixture of soil bacteria is not the way to achieve strong effects on plant growth promotion. Instead, further research is needed to find the optimal consortia of *A. castellanii* and nitrogen-fixing plant growth-promoting bacteria, eventually with additional nutrients for protists to provide, for example, soil-available phosphate for barley plants. This research could lead to reduced fertilizer use in organic barley production.

## supplementary material

10.1099/acmi.0.000761.v3 Uncited Supplementary Material 1.

## Abbreviations

DW: dry weight
IAA: indole-3-acetic acid
PGPB: plant growth-promoting bacteria
RI: relative increase

## References

[1] Sacharow J, Ratering S, Schneider B, Österreicher Cunha-Dupont A, Schnell S (2024). *Figshare*.

[2] Suarez C, Cardinale M, Ratering S, Steffens D, Jung S (2015). Plant growth-promoting effects of *Hartmannibacter diazotrophicus* on summer barley (*Hordeum vulgare* L.) under salt stress. Appl Soil Ecol.

[3] Yuan J, Ruan Y, Wang B, Zhang J, Waseem R (2013). Plant growth-promoting rhizobacteria strain *Bacillus amyloliquefaciens* NJN-6-enriched bio-organic fertilizer suppressed Fusarium wilt and promoted the growth of banana plants. J Agric Food Chem.

[4] Behrooz A, Vahdati K, Rejali F, Lotfi M, Sarikhani S (2019). *Arbuscular mycorrhiza* and plant growth-promoting bacteria alleviate drought stress in walnut. Horts.

[5] Krome K, Rosenberg K, Bonkowski M, Scheu S (2009). Grazing of protozoa on rhizosphere bacteria alters growth and reproduction of *Arabidopsis thaliana*. Soil Biol Biochem.

[6] Azam F, Fenchel T, Field JG, Gray JS, Meyer-Reil LA (1983). The ecological role of water-column microbes in the sea. Mar Ecol Prog Ser.

[7] Clarholm M (1985). Interactions of bacteria, protozoa and plants leading to mineralization of soil nitrogen. Soil Biol Biochem.

[8] Dumack K, Feng K, Flues S, Sapp M, Schreiter S (2022). What drives the assembly of plant-associated protist microbiomes? Investigating the effects of crop species, soil type and bacterial microbiomes. Protist.

[9] Geisen S, Mitchell EAD, Adl S, Bonkowski M, Dunthorn M (2018). Soil protists: a fertile frontier in soil biology research. FEMS Microbiol Rev.

[10] Triplett LR, Taerum SJ, Patel RR (2023). Protists at the plant-bacterial interface: impacts and prospective applications. Physiol Mol Plant Pathol.

[11] Jentschke G, Bonkowski M, Godbold DL, Scheu S (1995). Soil protozoa and forest tree growth: non-nutritional effects and interaction with mycorrhizae. Biol Fertil Soils.

[12] Bonkowski M, Brandt F (2002). Do soil protozoa enhance plant growth by hormonal effects?. Soil Biol Biochem.

[13] Bonkowski M (2004). Protozoa and plant growth: the microbial loop in soil revisited. New Phytol.

[14] Asiloglu R, Shiroishi K, Suzuki K, Turgay OC, Murase J (2020). Protist-enhanced survival of a plant growth promoting rhizobacteria, *Azospirillum* sp. B510, and the growth of rice (*Oryza sativa* L.) plants. Appl Soil Ecol.

[15] Hawxhurst CJ, Micciulla JL, Bridges CM, Shor M, Gage DJ (2023). Soil protists can actively redistribute beneficial bacteria along medicago truncatula roots. Appl Environ Microbiol.

[16] Chandarana KA, Pramanik RS, Amaresan N (2022). Interaction between ciliate and plant growth promoting bacteria influences the root structure of rice plants, soil PLFAs and respiration properties. Rhizosphere.

[17] Vestergård M, Bjørnlund L, Henry F, Rønn R (2007). Decreasing prevalence of rhizosphere IAA producing and seedling root growth promoting bacteria with barley development irrespective of protozoan grazing regime. Plant Soil.

[18] Ekelund F, Saj S, Vestergård M, Bertaux J, Mikola J (2009). The “soil microbial loop” is not always needed to explain protozoan stimulation of plants. Soil Biol Biochem.

[19] Zhang W, Lin Q, Li G, Zhao X (2022). The ciliate protozoan *Colpoda cucullus* can improve maize growth by transporting soil phosphates. J Integr Agric.

[20] Gao M, Xiong C, Tsui CKM, Cai L (2024). Pathogen invasion increases the abundance of predatory protists and their prey associations in the plant microbiome. Mol Ecol.

[21] Guo S, Jiao Z, Yan Z, Yan X, Deng X (2024). Predatory protists reduce bacteria wilt disease incidence in tomato plants. Nat Commun.

[22] Kuppardt A, Fester T, Härtig C, Chatzinotas A (2018). Rhizosphere protists change metabolite profiles in *Zea mays.*. Front Microbiol.

[23] Berlinches de Gea A, Li G, Chen JO, Wu W, Kohra A (2023). Increasing soil protist diversity alters tomato plant biomass in a stress-dependent manner. Soil Biol Biochem.

[24] Kreuzer K, Adamczyk J, Iijima M, Wagner M, Scheu S (2006). Grazing of a common species of soil protozoa (*Acanthamoeba castellanii*) affects rhizosphere bacterial community composition and root architecture of rice (*Oryza sativa* L.). Soil Biol Biochem.

[25] Weidner S, Latz E, Agaras B, Valverde C, Jousset A (2017). Protozoa stimulate the plant beneficial activity of rhizospheric pseudomonads. Plant Soil.

[26] Chandarana KA, Pramanik RS, Amaresan N (2022). Predatory activity of *Acanthamoeb*a sp genotype T4 on different plant growth-promoting bacteria and their combined effect on rice seedling growth. Eur J Protistol.

[27] Garcia J, Barker DG, Journet EP (2006). Medicago Truncatula Handbook.

[28] Crane-Droesch A, Abiven S, Jeffery S, Torn MS (2013). Heterogeneous global crop yield response to biochar: A meta-regression analysis. Environ Res Lett.

[29] Kandeler E, Gerber H (1988). Short-term assay of soil urease activity using colorimetric determination of ammonium. Biol Fert Soils.

[30] R Core Team (2020.). R: A language and environment for statistical computing (4.0.3). R foundation for statistical computing.

[31] Shapiro SS, Wilk MB (1965). An analysis of variance test for normality (complete samples). Biometrika.

[32] Bartlett MS (1937). Properties of sufficiency and statistical tests. Statistician.

[33] Girden ER (1992). ANOVA: Repeated Measures.

[34] Student (1908). The probable error of a mean. Biometrika.

[35] Kruskal WH, Wallis WA (1952). Use of ranks in one-criterion variance analysis. J Am Stat Assoc.

[36] Wilcoxon F (1945). Individual comparisons by ranking methods. Biometr Bull.

[37] Benjamini Y, Hochberg Y (1995). Controlling the false discovery rate: A practical and powerful approach to multiple testing. J R Stat Soc Ser B.

[38] Origin (2017). OriginLab Corporation.

[39] Patil I (2021). Visualizations with statistical details: the “ggstatsplot” approach. J Open Sour Softw.

[40] Krome K, Rosenberg K, Dickler C, Kreuzer K, Ludwig-Müller J (2010). Soil bacteria and protozoa affect root branching via effects on the auxin and cytokinin balance in plants. Plant Soil.

[41] Chandarana KA, Amaresan N (2023). Predation pressure regulates plant growth promoting (PGP) attributes of bacterial species. J Appl Microbiol.

[42] Su YH, McGrath SP, Zhao FJ (2010). Rice is more efficient in arsenite uptake and translocation than wheat and barley. Plant Soil.

