# Supplementary material for: Acanthamoeba castellanii alone is not a growth promoter for Hordeum vulgare
Source: Access Microbiol. 2024 Aug 1;6(8):000761.v3. doi: 10.1099/acmi.0.000761.v3 (PMC11293846; doi:10.1099/acmi.0.000761.v3)
Supplement: Uncited Supplementary Material 1. [file acmi-6-00761-s001.pdf]

Acanthamoeba castellanii alone is not a growth promoter for _Hordeum vulgare_

Julia Sacharow [a,b], Stefan Ratering [a], Bellinda Schneider [a], Alessandra Österreicher Cunha-Dupont [a] and Sylvia Schnell [a]

Institute of Applied Microbiology, IFZ, Justus-Liebig-University Giessen, 35392 Giessen, Germany [a]

Corresponding author [b]

Julia Sacharow

Email: Julia.Sacharow@umwelt.uni-giessen.de

Keywords: *Acanthamoeba castellanii*, Plant growth promoting, Soil protozoa, Root architecture, Soil food web, *Hordeum vulgare*.

Supplementary material figure 1: *Acanthamoeba castelanii* (A) trophozoite and (B) several cysts (marked with arrows) from the pouches of treatment 1) *A. castellanii* with soil bacteria after 21 days viewed under the microscope.

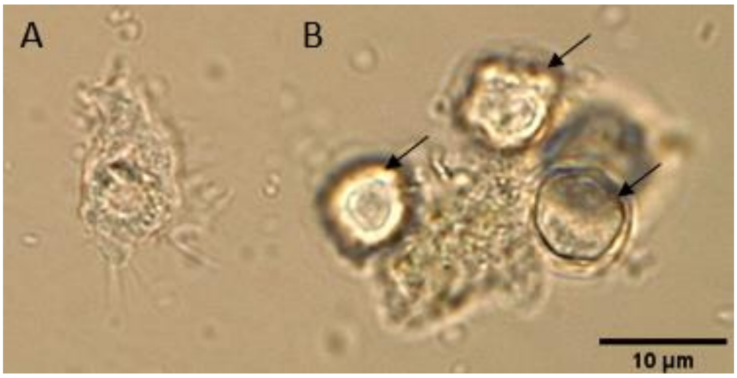

Supplementary material table 1: Standard deviation σ of root growth (length in cm, surface in cm^2 and volume in cm^3) of the barley plants at the different sampling points at 7, 14 and 21 days after the start of the experiment for treatments: 1) *A. castellanii* with soil bacteria, 2) *A. castellanii*, 3) Supernatant of *A. castellanii* with soil bacteria, 4) Supernatant of *A. castellanii*, 5) Volvic water with soil bacteria and 6) Volvic water.

	**First sampling**			**Second sampling**			**Third sampling**		
	Length	Surface	Volume	Length	Surface	Volume	Length	Surface	Volume
**1) *A. castellanii* with soil bacteria**	17.20	1.96	0.02	34.87	2.68	0.02	66.04	5.62	0.04
**2) *A. castellanii***	14.48	3.05	0.06	31.22	2.69	0.02	72.09	5.57	0.04
**3) Supernatant of *A. castellanii* with soil bacteria**	20.41	2.85	0.04	47.92	3.97	0.04	58.10	4.34	0.03
**4) Supernatant of *A. castellanii***	12.29	1.74	0.03	36.91	2.87	0.03	96.01	7.95	0.09
**5) Volvic water with soil bacteria**	18.49	2.71	0.03	55.88	4.14	0.03	67.81	5.22	0.03
**6) Volvic water**	16.20	2.57	0.04	41.15	3.78	0.03	65.04	6.89	0.06

Supplementary material table 2: *p*-values from various post-hoc tests ($p < 0.05$) of root length, root surface and root volume. The treatment 1) *A. castellanii* with soil bacteria is compared to the control treatments 3) Supernatant of *A. castellanii* with soil bacteria and 5) Volvic water with soil bacteria. The treatment 2) *A. castellanii* is compared to the control treatments 4) Supernatant of *A. castellanii* and 6) Volvic water at the three different sampling points. The two treatments 1) *A. castellanii* with soil bacteria and 2) *A. castellanii* are also compared. – No *p*-value needed

	2) *A. castellanii*	3) Supernatant of *A. castellanii* with soil bacteria	4) Supernatant of *A. castellanii*	5) Volvic water with soil bacteria	6) Volvic water
	**First sampling**				
**1) *A. castellanii* with soil bacteria - root length**	1.00	0.87	-	0.87	-
**1) *A. castellanii* with soil bacteria - root surface**	0.66	0.83	-	0.66	-
**1) *A. castellanii* with soil bacteria - root volume**	0.62	0.87	-	0.53	-
**2) *A. castellanii* - root length**	-	-	0.87	-	1.00
**2) *A. castellanii* - root surface**	-	-	0.66	-	0.66
**2) *A. castellanii* - root volume**	-	-	0.62	-	0.53
	**Second sampling**				
**1) *A. castellanii* with soil bacteria - root length**	0.86	0.86	-	0.86	-
**1) *A. castellanii* with soil bacteria - root surface**	0.26	0.40	-	0.99	-
**1) *A. castellanii* with soil bacteria - root volume**	0.05	0.12	-	0.72	-
**2) *A. castellanii* - root length**	-	-	1.00	-	0.86
**2) *A. castellanii* - root surface**	-	-	0.82	-	0.75
**2) *A. castellanii* - root volume**	-	-	0.39	-	0.56
	**Third sampling**				
**1) *A. castellanii* with soil bacteria - root length**	0.90	0.99	-	0.99	-
**1) *A. castellanii* with soil bacteria - root surface**	0.97	0.97	-	0.97	-
**1) *A. castellanii* with soil bacteria - root volume**	0.89	0.94	-	0.89	-
**2) *A. castellanii* - root length**	-	-	0.99	-	0.99
**2) *A. castellanii* - root surface**	-	-	0.97	-	0.97
**2) *A. castellanii* - root volume**	-	-	0.89	-	0.89

Supplementary material table 3: Standard deviation σ of leaf growth (length in cm) of the barley plants at the different sampling points at 7, 14 and 21 days after the start of the experiment for treatments: 1) *A. castellanii* with soil bacteria, 2) *A. castellanii*, 3) Supernatant of *A. castellanii* with soil bacteria, 4) Supernatant of *A. castellanii*, 5) Volvic water with soil bacteria and 6) Volvic water.

	First sampling	Second sampling	Third sampling
**1) *A. castellanii* with soil bacteria**	1.64	2.10	2.16
**2) *A. castellanii***	2.17	1.68	2.26
**3) Supernatant of *A. castellanii* with soil bacteria**	1.98	1.86	1.72
**4) Supernatant of *A. castellanii***	1.35	2.50	3.25
**5) Volvic water with soil bacteria**	1.67	2.73	2.50
**6) Volvic water**	1.50	2.61	2.24

Supplementary material table 4: *p*-values from various post-hoc tests ($p < 0.05$) of leaf length. The treatment 1) *A. castellanii* with soil bacteria is compared to the control treatments 3) Supernatant of *A. castellanii* with soil bacteria and 5) Volvic water with soil bacteria as well as the treatment 2) *A. castellanii*. The treatment 2) *A. castellanii* is compared to the control treatments 4) Supernatant of *A. castellanii* and 6) Volvic water at the three different sampling points. – No *p*-value needed.

	**2) *A. castellanii***	**3) Supernatant of *A. castellanii* with soil bacteria**	**4) Supernatant of *A. castellanii***	**5) Volvic water with soil bacteria**	**6) Volvic water**
**1) *A. castellanii* with soil bacteria – first sampling**	0.99	0.99	-	0.99	-
**1) *A. castellanii* with soil bacteria – second sampling**	0.80	0.78	-	0.24	-
**1) *A. castellanii* with soil bacteria – third sampling**	0.93	0.93	-	0.93	-
**2) *A. castellanii* – first sampling**	-	-	0.99	-	0.99
**2) *A. castellanii* – second sampling**	-	-	0.95	-	0.95
**2) *A. castellanii* – third sampling**	-	-	0.93	-	0.93

Important steps of the R code

```
# Importing the data
Data <- read.table ("path_to_generated_data", sep="\t", dec=",",header=T)


# Analysis of the results – Normal distribution
Model <- aov(Choosen_sampling~Treatment, data= Data)
residuen <- residuals(Model)
qqnorm(residuen)
qqline(residuen)
shapiro.test(residuen)


# Analysis of the results – Homogeneity of variances
bartlett.test(Data$Choosen_sampling~Data$Treatment)


# Analysis of the results – Pairwise comparison
# Normal distribution and homogeneity of variances
pairwise.t.test(Data$Choosen_sampling,Data$Treatment,p.adjust="BH")

# No normal distribution
pairwise.wilcox.test(Data$Choosen_sampling,HV$Treatment, p.adjust="BH")

# Normal distribution but no homogeneity of variances
pairwise.t.test(Data$Choosen_sampling,HV$Treatment,p.adjust="BH",var.equal=FALSE)


# Violin plots
library("ggstatsplot")
violinplot <- ggbetweenstats(data = Data, x = Treatment, y = Choosen_sampling, plot.type = "violin")+scale_color_manual(values = c("darkgreen", "hotpink", "blue", "yellow", "red", "lightblue"))
VIOLIN <- violinplot+labs(x = " Treatment ",y = " Choosen_sampling",title = "Plot")
VIOLIN
```
